# Using Aptamers as a Novel Method for Determining GnRH/LH Pulsatility

**DOI:** 10.3390/ijms21197394

**Published:** 2020-10-07

**Authors:** Chioma Izzi-Engbeaya, Ali Abbara, Anthony Cass, Waljit S Dhillo

**Affiliations:** 1 Section of Endocrinology and Investigative Medicine, Imperial College London, London W12 0NN, UK; c.izzi@imperial.ac.uk (C.I.-E.); ali.abbara@imperial.ac.uk (A.A.); 2 Department of Endocrinology, Imperial College Healthcare NHS Trust, London W2 1NY, UK; 3 Department of Chemistry, Imperial College London, London SW7 2AZ, UK; t.cass@imperial.ac.uk

**Keywords:** aptamer, synthetic oligonucleotides, gonadotropin-releasing hormone (GnRH), luteinizing hormone (LH), pulsatility

## Abstract

Aptamers are a novel technology enabling the continuous measurement of analytes in blood and other body compartments, without the need for repeated sampling and the associated reagent costs of traditional antibody-based methodologies. Aptamers are short single-stranded synthetic RNA or DNA that recognise and bind to specific targets. The conformational changes that can occur upon aptamer–ligand binding are transformed into chemical, fluorescent, colour changes and other readouts. Aptamers have been developed to detect and measure a variety of targets in vitro and in vivo. Gonadotropin-releasing hormone (GnRH) is a pulsatile hypothalamic hormone that is essential for normal fertility but difficult to measure in the peripheral circulation. However, pulsatile GnRH release results in pulsatile luteinizing hormone (LH) release from the pituitary gland. As such, LH pulsatility is the clinical gold standard method to determine GnRH pulsatility in humans. Aptamers have recently been shown to successfully bind to and measure GnRH and LH, and this review will focus on this specific area. However, due to the adaptability of aptamers, and their suitability for incorporation into portable devices, aptamer-based technology is likely to be used more widely in the future.

## 1. Introduction

Gonadotropin-releasing hormone (GnRH) is decapeptide secreted into the hypophyseal portal system by specialised hypothalamic neurons, to reach its site of action at the pituitary gland. GnRH acts on pituitary gonadotropes to stimulate the production and secretion of gonadotropins (luteinizing hormone (LH) and follicle stimulating hormone (FSH)) [1]. LH and FSH stimulate the secretion of sex steroids from the gonads (primarily testosterone from the testes and oestrogen/progesterone from the ovaries) [2]. Apart from during the pre-ovulatory phase of the menstrual cycle, GnRH and LH secretion are inhibited by sex steroids (i.e., negative feedback) [3]. However, during the pre-ovulatory phase of the menstrual cycle, oestrogen exerts positive feedback on GnRH and LH secretion, leading to increased LH secretion and the production of the LH surge required for ovulation [3].

GnRH, LH and FSH all exhibit pulsatile secretory patterns, which are pivotal to their biological functions [3], including stimulation of gonadal sex steroid secretion. GnRH has a short half-life of 2-8 min in the peripheral circulation [4], with very low levels (i.e., ≤5pg/ml) detected in plasma [5]. Therefore, it is challenging to measure GnRH in the peripheral circulation. As LH pulses are temporally coupled with GnRH pulses and LH is stable in the blood [6], LH levels and pulses are commonly measured and used as the clinical gold standard surrogate markers of GnRH secretion.

GnRH and LH pulsatility change during different reproductive stages and at different phases of the menstrual cycle. Pre-pubertally, LH levels are low and very few LH pulses are detectable. However, as puberty progresses, LH pulse frequency increases in response to increased GnRH pulsatility [7]. In adulthood, LH pulses occur approximately every 2 h in healthy men [8]. In healthy women, LH pulses occur every 1-2 h in the follicular phase and every 4 h in the luteal phase [2]. When reproductive senescence occurs in menopausal women, LH levels are markedly elevated (resulting from the absence of negative feedback due to oestrogen deficiency), and approximately one LH pulse occurs each hour [9].

Reproductive pathology is characterised by alterations in the normal patterns of GnRH and LH pulsatility. In hypogonadotropic hypogonadism, LH, FSH and sex steroid levels are abnormally low, with few or absent pulses. This may occur due to genetic defects giving rise to Congenital Hypogonadotropic Hypogonadism (CHH—e.g., *ANOS1* gene variations in Kallman’s Syndrome) [10]. However, in adolescents who have constitutional delay of growth and puberty (CDGP), the pattern of LH, FSH and sex steroids is indistinguishable from the pattern seen in CHH. Whilst children with CDGP will eventually progress through puberty, people with CHH require long-term hormonal supplementation. Therefore, it is important to be able to distinguish between these conditions, but there is still significant diagnostic uncertainty using current approaches [10]. Evaluation of LH pulsatility may help reduce this uncertainty, as patients with CHH have been shown to lack the sleep-entrained increase in LH pulse amplitude and number present in 75% of pre-pubertal children [11]; and in the small proportion of patients with CHH that have detectable pulsatile LH secretion (25% in one series of 78 men), the LH pulses are abnormal (i.e., low amplitude and/or low frequency) [12].

Acquired hypogonadotropic hypogonadism can be caused by pituitary dysfunction due to trauma, surgery, cranial irradiation, obesity, opioids, infiltrative and/or autoimmune disease. Additionally, a type of hypogonadotropic hypogonadism (termed hypothalamic amenorrhea in women) may also occur due to stress, weight loss and/or an extended period of intense physical activity, and this reproductive disorder occurs in 4% of pre-menopausal women [13]. Abnormal LH pulsatility is also present in Polycystic Ovarian Syndrome (PCOS, which affects up to 13% of women of reproductive age) and is characterised by at least two of the following features: hyperandrogenism, polycystic ovarian morphology on ultrasound and/or oligomenorrhea/amenorrhea [14]. Furthermore, it has been recognised that these conditions may co-exist with a subset of women exhibiting clinical and/or biochemical features of both hypothalamic amenorrhea and PCOS [15]. Women with hypothalamic amenorrhea have low LH levels and reduced LH pulsatility [16], whereas women with PCOS have high basal LH levels and increased pulsatility [17]. However, despite the valuable information provided by the assessment of hormone pulsatility that can be used to aid diagnosis of reproductive disorders, it is not performed in routine clinical practice (for the reasons outlined below), and thus, LH pulse assessment is currently only performed in research centres. 

The detection and quantification of hormones (e.g., LH) have been reliant on antibody-based techniques for many decades. However, problems with these methods include suboptimal inter-assay reliability and reproducibility, high cost of reagents, high sample volumes required (limiting the number of samples that can be taken from a single subject) and the time-consuming nature of antibody-based assays [18]. To overcome these problems, alternative methods of analyte detection and quantification have been developed. One such method is based on the use of aptamers (i.e., synthetic ligand-binding oligonucleotides). In line with advancements in this technology, the development of aptamer-based assays has accelerated over the last two decades, such that their use is likely to become more widespread. 

In this review, we highlight the major types of aptamers in development and outline possible applications for aptamer-based technology in the assessment of GnRH/LH pulsatility.

## 2. Aptamers

**Figure 1:**
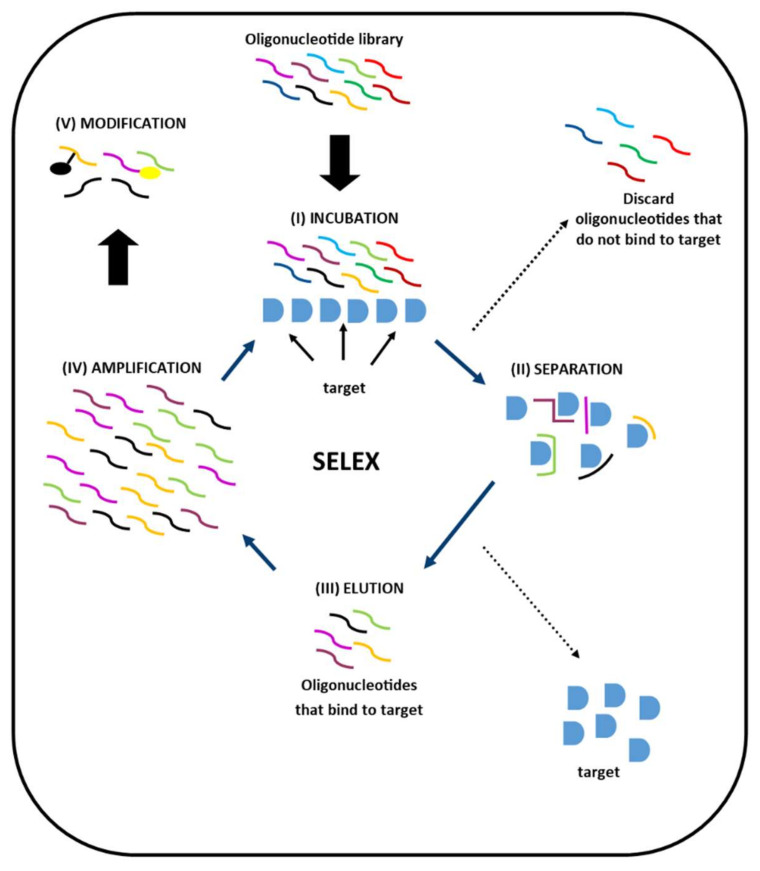
Summary of systematic evolution of ligands by exponential enrichment (SELEX) procedure for generation of aptamers for a specific target ligand. (I) Oligonucleotides from a library are incubated with the target. (II) Following incubation, oligonucleotides that do not bind to the target are separated from the bound oligonucleotide-target complexes and discarded. (III) Oligonucleotides that bind to the target are eluted. (IV) The eluted oligonucleotides are then amplified and incubated with the target. (I) The cycle is then repeated several times, with increased stringency of the elution conditions during subsequent cycles, to produce an enriched pool of the best candidate oligonucleotides. (V) After the last cycle, the selected oligonucleotides are then assessed, and the sequences with the highest binding affinity are selected and synthesised. These sequences can then be modified by addition of groups to the 3′-end to reduce their susceptibility to nucleases, alteration of nucleotide bases and/or generation of spigelmers (i.e., mirror-image isomers).

Aptamers are single-stranded DNA or RNA oligonucleotides that are selected to recognise and bind to specific ligands. Currently, most aptamers are developed using systematic evolution of ligands by exponential enrichment (SELEX) [19,20]. In SELEX, an oligonucleotide library is generated or purchased from commercial providers. The library is incubated with the target ligand, and sequences that do not bind to the ligand are discarded. Sequences that are bound to the ligand are then dissociated from the ligand, collected and amplified using polymerase chain reactions (PCR), preceded by reverse transcription for RNA aptamers and followed by single strand DNA generation for DNA aptamers. The cycle of ligand-binding, removal of unbound sequences, collection and amplification of bound sequences is repeated to obtain significant quantities of the sequences that bind efficiently to the ligand. Assays are performed using the ligand-binding sequences, and the highest-performing sequence(s) is (are) identified and synthesised (Figure 1). 

Post-SELEX, modifications of the aptamer structure can be employed to improve stability (e.g., by capping the 3′-end of the oligonucleotide with inverted thymidine or biotin to reduce its susceptibility to 3′-exonuclease [21,22]), increase the half-life of the aptamer in vivo (e.g., by conjugation of polyethylene glycol (PEG) to the 5′-end [23] to reduce the rate of renal clearance) and enhance ligand-binding affinity (e.g., by adding amino groups to the oligonucleotide sequence [24] and/or modifying nucleotide bases within the sequence [25]). More recently, other types of modified aptamers have been developed, with increased resistance to degradation by nucleases and/or increased binding affinities, including spiegelmers (oligonucleotide sequences consisting of L-(deoxy)ribonucleic acids that are synthetic mirror-image isomers of naturally-occurring D-(deoxy)ribonucleic acids) [26]; circular aptamers [27]; and multimers (i.e., aptamers that have the ability to bind to more than one region of their ligands, which improve the aptamers’ sensitivity and specificity) [28,29].

**Figure 2:**
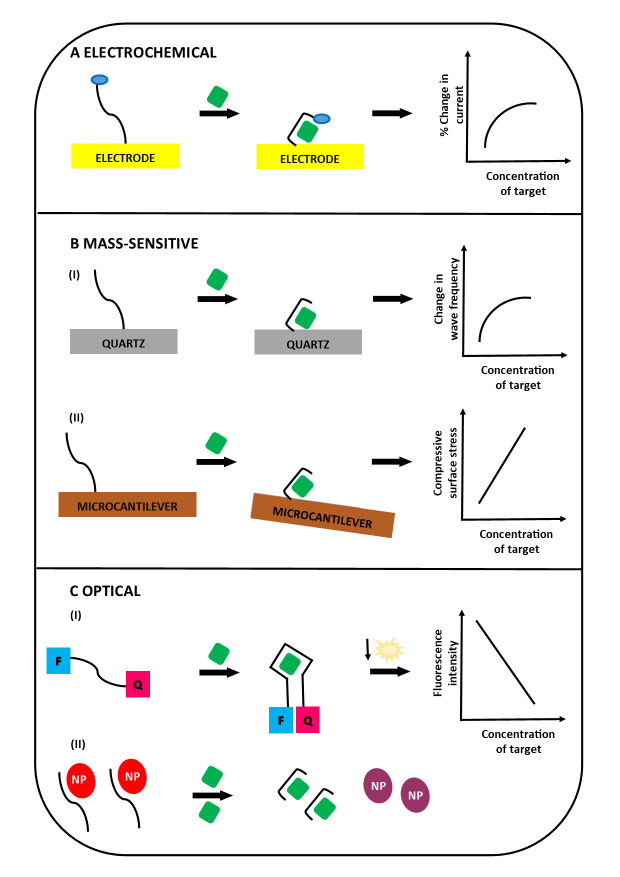
Examples of electrochemical, mass-sensitive and optical aptamer biosensors. **A**—The aptamer is attached to an electrode at one end with an electrochemical reporter (blue oval) covalently linked to the other end. Binding of the aptamer to its target ligand (green rectangle) results in a change in its shape, which alters the distance between the electrochemical reporter and the electrode. Consequently, the electron transfer rate between the electrochemical reporter and the electrode changes, producing a change in the current. **B**—(I) In a mass-sensitive biosensor, the aptamer is attached to a quartz surface, and aptamer–ligand binding results in a change in wave frequency. (II) In a different version of the mass-sensitive biosensor, the aptamer is immobilised on the surface of a microcantilever. Aptamer–ligand binding produces a change in the compressive stress at the microcantilever surface. **C**—(I) In an optical biosensor, a fluorophore (F) is attached to one end of the aptamer and a corresponding quencher (Q) is attached to the other end. Aptamer–ligand binding changes the shape of the aptamer, which brings the fluorophore and quencher closer to together. This results in a reduction in the fluorescence intensity. (II) In a different type of optical biosensor, gold nanoparticles (NP) associate with the unbound aptamers. Aptamer–ligand binding causes the nanoparticles to dissociate from the aptamer. The dissociated nanoparticles aggregate, which (in the presence of salt—NaCl) produces a visible colour change.

The ability of an aptamer to bind to its target depends on its three-dimensional structure [19,20]. Additionally, the aptamer–ligand interaction can produce a marked conformational change in the aptamer [30], which can be used to provide a bioassay read-out using electrochemical, mass-sensitive and optical methodologies (Figure 2).

Electrochemical aptamer sensors consist of aptamers immobilised on an electrode at one end and attached to an electrochemical reporter (such as methylene blue [30,31], ferrocene [32], ferrocene-functionalised polyelectrolyte poly(3)-alkoxy-4-methylthiophene [33] or ruthenium (III) hexamine [34]) on the other end. Aptamer–ligand binding alters the shape of the aptamer, resulting in a change in the electron transfer rate between the electrochemical reporter and the electrode. This produces a change in the electrical current, the magnitude of which is proportional to the amount of ligand present, and this change in current is measured by a potentiostat. Mass-sensitive aptamer sensors measure analytes by detecting changes in the refractive index at a surface [35], changes in resonance frequency of quartz crystals [36,37] or altered degree of bending between microcantilevers [38] produced by aptamer–ligand binding. In fluorescent optical systems, binding of the ligand to an aptamer labelled with a fluorophore at one end and a quencher at the other, results in increased distance between the fluorophore and the quencher and enhancement of a fluorescent signal [39,40]. In a variant of the fluorescent system, the unbound aptamer’s structure keeps the fluorophore and quencher apart until the conformational change caused by aptamer–ligand binding brings the fluorophore and quencher close together, resulting in a reduction in fluorescence intensity [41,42]. In a different type of fluorescent system, pyrene moieties at each end of an aptamer form an exciplex and fluoresce when aptamer–ligand binding occurs [43]. Another commonly used fluorescent system is the donor–acceptor system, in which the aptamer is labelled with Cy3 (donor) at one end and Cy5 (acceptor) at the other end, and detectable fluorescence resonance energy transfer (FRET) occurs between the donor–acceptor pair after the aptamer binds to its ligand [44]. In colorimetric optical systems, aptamers dissociate from the nanoparticles following aptamer–ligand binding and change colour in the presence of sodium chloride (NaCl) and/or heat [45,46]; or an enzymatic colour change is produced by aptamer–ligand binding following exposure to UV light [47,48].

Currently, the diagnosis of many conditions is made using antibody-based methods of detection and/or quantification of biomarkers specific to the condition of interest, such as the measurement of LH, FSH and testosterone using chemiluminescent immunoassays to determine if hypogonadism is primary (due to testicular failure) or secondary (due to pituitary or hypothalamic dysfunction). However, variability between batches of the same antibody can produce variation in results within the same experimental setting [49], as well as the potential for cross-reactivity with other analytes reducing the specificity of an antibody (with one study reporting that only 56% of the 109 commercially available antibodies tested displayed 100% specificity for their peptide targets [50]). Suboptimal antibody specificity increases the likelihood of obtaining false positive results, with resulting significant adverse consequences for patients (such as additional investigations, unnecessary treatment and/or psychological harm [51]). Thus, there is a need to explore alternative methods of analyte detection and measurement. 

Aptamers possess several properties that may prove to be advantageous. For instance, it typically takes months to produce antibodies whilst aptamers can be produced in weeks. The production of aptamers does not require the use of animals [30], and their production can be rapidly upscaled, which could lead to reduced costs. Aptamers can detect metal ions [52] and small molecules [52,53,54,55] that cannot be recognised by antibodies. Furthermore, certain (i.e., electrochemical) aptamer-based sensors do not require additional reagents [30]; therefore, readouts are obtained more quickly, and multiple measurements can be made periodically and/or continuously over an extended period of time.

Aptamers can be modified (more readily than antibodies) to increase their specificity, ligand affinity, half-lives and/or facilitate their incorporation into sensor devices [56,57]. Recently described aptamer-based devices include a lateral flow strip for the detection of hepatitis C core antigen in serum (with a colour-change produced by a positive result visible to the naked eye within 10 min) [58], a microfluidic paper device for detection of aflatoxin B1 in milk [59] and standard blood glucose meters have been adapted for quantification of interferon-γ [60] and cocaine in human serum [61] using aptamers. Additionally, aptamer-based devices have also been used for in vivo measurement of cocaine in different regions of rat brains using modified electrodes [62] and for in vivo measurement of antibiotics in ambulatory rats using implanted sensors [63].

Furthermore, aptamer microarray platforms can be used to detect a large number of protein analytes in biological samples simultaneously (which is currently only possible to a limited extent with antibody-based methodologies). In these platforms, ~1000 Slow Off-rate Modified Aptamers (SOMAmers—i.e., aptamers with slow ligand dissociation rates that are selected for different proteins), which carry both a 5′-photocleavable biotin tag and a 3′-fluorescent label, are incubated with samples (e.g., plasma, serum or cell lysates). The SOMAmer-protein complexes are captured onto streptavidin coated beads (via streptavidin-biotin binding), and unbound proteins are washed away. The proteins that are bound to SOMAmers are then tagged with non-photocleavable biotin. Ultraviolet light is used to separate the photocleavable biotin from the SOMAmers, and the photocleavable biotin remains attached to the streptavidin beads. The SOMAmer-protein complexes are then immobilised on new beads via streptavidin binding to the non-photocleavable biotin and free SOMAmers are washed away. The proteins are then dissociated from the SOMAmers by raising the pH of the solution. The SOMAmers are then hybridized to a microarray of single-stranded DNA probes complementary to the SOMAmers’ oligonucleotide sequences. When the microarray is scanned, the hybridized SOMAmers (and, therefore, the proteins detected by the SOMAmers in the samples) are then quantified by fluorescence of the 3′ label. SOMAmer platforms have been used to identify potential chronic kidney disease biomarkers in human plasma [64]; identify novel Duchenne Muscular Dystrophy biomarkers in mouse serum [65]; discover differential protein expression patterns between tumour and non-tumour tissue in hepatic tissue resected from people with hepatocellular cancer [66]; and identify proteins in plasma subsequently used to develop and validate a prospective risk score for patients with ischaemic heart disease [67].

Thus, the versatility of aptamer-based techniques and the many advantages they have over antibody-based methodologies make them suitable for the detection and quantification of GnRH and LH in vitro and in vivo.

## 3. GnRH Aptamers

Several GnRH aptamers have been described, which bind to GnRH in vitro (in non-buffered solutions, cell culture and urine) and in vivo (in rats and rabbits). Using SELEX followed by solid phase synthesis utilising chiral isomeric nucleotides, a high-affinity GnRH RNA spiegelmer and a high-affinity GnRH DNA spiegelmer were generated with equilibrium dissociation constants (*K*_D_) of approximately 45 nM [68]. These aptamers bound preferentially to GnRH, with minimal binding to buserelin (a GnRH analog) and chicken luteinizing-releasing hormone (LHRH, which differs from GnRH by only a single amino acid), and did not bind to vasopressin and oxytocin (i.e., neuropeptides that are structurally different from GnRH) at concentrations ranging from 0.1-1000 µM [68]. Both spiegelmers prevented GnRH-mediated activation of its receptor (as measured by calcium ion release) in Chinese hamster ovary (CHO) cells transfected with the human GnRH receptor, with IC_50_ values of ~200 nM for the RNA spieglemer and ~50 nM for the DNA aptamer [68].

A different DNA spieglemer with a higher affinity to GnRH (*K*_D_ = 20 nM), called NOX 1255, and a pegylated form of NOX 1255 (i.e., NOX 1257) inhibited GnRH-mediated calcium ion release (with an IC_50_ value of 20 nM) in CHO cells transfected with the human GnRH receptor [54]. NOX 1255 and NOX 1257 are highly specific for GnRH as there was no inhibition of buserelin activity when these aptamers were co-incubated with buserelin in cultures of CHO cells [54]. In vivo, in castrated male rats, subcutaneous administration of NOX 1255 suppressed LH levels by 67% for up to 6 h, whilst intravenous NOX 1257 suppressed LH to pre-castration levels for 24 h [56]. Additionally, neither repeated dosing of NOX 1255 or NOX 1257 over 14 weeks elicited an immune response in rabbits [56], a species that is commonly used to generate antibodies used in antibody-based assays [69].

These studies demonstrate the high specificity and affinity of the selected GnRH aptamers. These GnRH aptamers selectively bind to GnRH, with little or no binding to structurally similar compounds (i.e., chicken LHRH and buserelin) and structurally dissimilar compounds (vasopressin and oxytocin). Furthermore, as conditions in vivo are different from the in vitro conditions under which aptamers are generated and tested, it is encouraging that the aptamers retained their activity in vivo. The extended suppression of LH produced by the PEG-modified aptamer, and the absent immunogenicity of NOX 1255 and NOX 1257 (in rabbits), makes these aptamers suitable agents for use in research (where GnRH-mediated pathways are being investigated) and as therapeutic agents (such as in assisted fertility cycles where endogenous LH suppression is required and in prostate cancer to produce androgen suppression). However, further safety and efficacy studies are required before the potential utility of these aptamers is realised. Encouragingly, a precedent for therapeutic aptamer use exists in the form of an anti-vascular endothelial growth factor aptamer that has been licensed for the treatment of macular degeneration [70,71].

More recently, a GnRH aptamer has been used successfully to measure GnRH in a prototype aptamer-nucleic acid sequence-based amplification (aptamer-NASBA) lab-on-a-chip device based on a 384-well microplate, which could be read by standard fluorescence microplate readers [72]. Samples containing sub-picomolar concentrations of GnRH were added to the aptamer-coated gold reaction surface of this device. After removing unbound GnRH by washing, anti-GnRH biotinylated antibodies were added followed by streptavidin, which bound to the biotinylated antibodies. The resulting aptamer-GnRH-biotinylated antibody-streptavidin sandwich was incubated with biotinylated single-stranded DNA that bound to the streptavidin at a different site. Following reverse transcription of the streptavidin-bound DNA to RNA, T7 RNA polymerase was used to amplify the RNA, thus producing an amplified fluorescent signal that was proportional to the concentration of GnRH in the sample. This aptamer-NASBA device could be used to measure multiple samples simultaneously in under 1 h, which would be useful when conducting pulsatility studies, where ideally sampling should be performed every 5-10 min for a minimum of 8 h [73]. NASBA assays can be performed with sample volumes as little as 5µl [74]; therefore, aptamer-NASBA assays would be suitable for pulsatility studies performed on rodents that have low circulating volumes of blood compared to larger mammals.

Blood sampling is invasive and can cause stress-induced cortisol release, which in turn suppresses GnRH and LH secretion and pulsatility [75,76]. The measurement of GnRH in urine may circumvent this problem. In greyhound dogs and humans, GnRH metabolites have been detected in urine, but intact GnRH in urine has only been detected after [but not prior to] administration of GnRH, using mass spectrometry [77,78]. Similarly, NOX 1255 (a GnRH spiegelmer) was unable to detect GnRH in the urine of healthy horses, but it detected GnRH one hour after GnRH administration [79]. Additionally, unlike the antibody-based assay, NOX 1255 did not detect the GnRH metabolite (GnRH_5-10_), indicating that the specificity of this aptamer for intact GnRH is preserved in urine. Collectively, these data suggest that NOX 1255 (and other GnRH aptamers) may be suitable for measurement of GnRH in urine after exogenous GnRH administration but not for measurement of endogenous GnRH levels.

## 4. LH Aptamers

It is not feasible to measure GnRH in the hypophyseal-portal circulation in humans (as this would require access to blood vessels at the base of the brain). Additionally, GnRH has a very short half-life in the peripheral circulation [4]. Therefore, as LH pulses are preceded by GnRH pulses in a one to one ratio [6,80], circulating LH is commonly measured and used as a gold standard surrogate marker of GnRH pulsatility in humans. However, standard methods of determining LH pulsatility require frequent manual blood sampling (i.e., every 10 min for a minimum of 8 h), with LH levels measured using antibody-based assays [8]. This is impractical in clinical settings and presents challenges in research settings as it is labour-intensive, a large number of samples and blood volumes are required (≥48 samples/8-h sampling period) and the associated costs are high (~25 US dollars/sample). Therefore, aptamer-based methods may prove to be a superior method of assessing LH pulsatility.

A recent ground-breaking study demonstrated the ability of an LH aptamer (coupled to a robotic electrochemical platform) to detect different patterns of LH secretion in stored undiluted serum samples from healthy pre-menopausal women (normal LH levels and pulsatility), post-menopausal women (high LH levels and increased pulsatility) and women with hypothalamic amenorrhea (low LH levels and reduced pulsatility) [30]. The electrochemical platform consisted of a potentiostat, laptop interface, programmable liquid-handling robot, wire electrodes functionalised with the LH aptamer and a 96-well-plate onto which samples were loaded [30]. Excellent correlation was reported between LH concentrations measured using the aptamer and levels measured with the traditional antibody-based assay performed using an in-hospital laboratory automated analyser (R^2^ = 0.94, *p* < 0.001), with pulsatility confirmed using Bayesian spectrum analysis [30].

This robotic electrochemical aptamer system was automated, reusable, low cost and highly specific for LH (as there was negligible cross-reactivity with FSH, which shares a common α-subunit with LH and which circulates at higher levels than LH in post-menopausal women [81]). These attributes confer significant advantages over traditional antibody-based LH measurement. Additionally, this LH aptamer system can be adapted by inclusion of other aptamers to enable measurement of multiple proteins (including hormones) in a single sample [64,82]; by integration of a wire electrode with an automated blood collection system for real-time measurements [83,84]; and/or by incorporation of a microneedle skin patch [85] to measure hormones in dermal interstitial fluid. Furthermore, other electrochemical aptamer platforms have been successfully adapted and used for real-time continuous monitoring of pre- and post-administration drug levels in venous blood of ambulatory rats for ≥5 h [63]. Therefore, further development of this novel robotic LH-aptamer system could enable continuous ambulatory real-time hormone sensing with increased accuracy and accessibility of pulsatility assessment of LH in different physiological and pathological conditions.

## 5. Potential Applications of GnRH/LH Aptamers

As aptamers have been used in conjunction with modified implantable electrodes (i.e., gold-coated electrodes functionalized with cocaine-specific aptamers) to measure cocaine in vivo in rat brains [62]; a similar approach could be used to measure GnRH in the hypothalamus in animals. This would provide invaluable information such as changes in GnRH pulsatility during different stages of reproductive maturity and senescence, and in rodent models of human reproductive orders such as PCOS (the most common reproductive disorder affecting pre-menopausal women [14]). Furthermore, incorporation of LH aptamers into intravenous or subcutaneous sensors would facilitate detailed characterisation of LH secretion and pulsatility in rodents, sheep and other mammals, which have been limited by factors such as blood volumes, technical challenges, as well as antibody-based assays with low specificity and inadequate sensitivity.

Furthermore, the development of the robotic LH-aptamer system described above [30] to include wireless connection to a portable transdermal sensor (similar to continuous glucose monitors currently in use for diabetes management [86]) would revolutionize reproductive endocrinology research, due to the reduced costs and quantity of (previously inaccessible) information this technology would enable researchers to obtain. In addition, the availability of an inexpensive portable method of assessing LH pulsatility would facilitate timely and accurate diagnosis of patients presenting to clinicians with reproductive disorders, particularly as currently these patients’ reproductive hormones are assessed using single timepoint blood tests. Thus, GnRH and LH aptamers could have significant and beneficial impacts on research and in healthcare.

## 6. Challenges

Although aptamer development has progressed over the last two decades, there are relatively few aptamer-based compounds currently in use in clinical research and healthcare. This might be due to differences in ligand binding and bioavailability of the aptamers in vitro (where they are generated) compared with in vivo use (where the temperature, pH and the presence of interfering factors such as lipids can influence the structure and function of the aptamers). Furthermore, in vivo, non-specific binding of the aptamer can occur, which can be difficult to predict during the process of aptamer selection and modification. However, cross-reactivity can be reduced by including negative selection cycles during SELEX to remove oligonucleotides that bind to proteins that are structurally similar to the target protein (e.g., oligonucleotides that bind to FSH were identified and discarded during negative selection SELEX cycles used to produce the LH aptamer discussed above [30]). Additionally, aptamers have been reported to have lower, similar or higher binding affinities compared with antibodies for a specific target [87]. Therefore, careful selection of the best candidate sequence and/or post-SELEX modification may be required to ensure that the binding affinity of an aptamer exceeds that of an antibody for a given target.

Modifications made to aptamers to improve their pharmacokinetic properties can also adversely affect their function. For instance, conjugation of PEG to an interleukin-17A RNA aptamer more than doubled the half-life of the aptamer in vivo [23]. However, significant titres of anti-PEG antibodies were detected in 37% of plasma samples from 377 healthy donors [88]. This may limit the widespread use of PEGylated aptamers as anti-PEG antibodies can reduce the efficacy of PEGylated aptamers [89], and these antibodies were identified as the cause of serious hypersensitivity reactions in clinical trials of an anticoagulant RNA PEGylated aptamer [90].

Another challenge is not all targets are suitable for aptamer generation. Unlike targets with positively charged groups, hydrophobic targets and targets containing phosphate groups [which are negatively charged] are more difficult to select appropriate aptamers for [91,92]. As GnRH and LH are not hydrophobic and do not contain phosphate groups, they are suitable aptamer targets as evidenced by the highly specific and sensitive GnRH and LH aptamers described above [30,56,68,77]. Additionally, initial aptamer generation costs are high. However, once a suitable aptamer has been generated, the production costs of the aptamer are likely to be lower than antibody-generation costs (e.g., <USD 50/g for aptamers supplied by Aptagen LLC [93] compared with ~USD 300/g for recombinant monoclonal antibodies produced using mammalian cell lines [94]).

There are currently no standardized aptamer selection protocols for a given target with over 20 different types of SELEX procedures described in the literature [95]. Additionally, the SELEX process can be slow (i.e., with 8-20 cycles performed), often yielding <15 candidate aptamers from an initial pool of 10^15^ molecules, which then require post-SELEX optimisation [30,68,96,97]. Overcoming these challenges will enable the full potential of this class of synthetic oligonucleotides to be realized for assessing GnRH/LH pulsatility and many other diverse applications.

## 7. Conclusions

Aptamer development is a rapidly expanding field with numerous potential research, diagnostic and therapeutic applications. Due to the many advantages of aptamers over traditional antibody-based detection and quantification methods, adoption of aptamer technology into routine use in research and healthcare settings may occur in the near future as technical challenges are overcome. Aptamer-based technology has the potential to be a powerful research tool, as well as to enable real-time, continuous, low cost ambulatory LH pulsatility assessments in clinical settings. This could transform clinical practice by providing more accurate diagnosis of reproductive disorders (currently based on single timepoint blood tests that do not assess LH pulsatility), and thus improve the management of these conditions.
